# Altered Kinematics of Facial Emotion Expression and Emotion Recognition Deficits Are Unrelated in Parkinson’s Disease

**DOI:** 10.3389/fneur.2016.00230

**Published:** 2016-12-14

**Authors:** Matteo Bologna, Isabella Berardelli, Giulia Paparella, Luca Marsili, Lucia Ricciardi, Giovanni Fabbrini, Alfredo Berardelli

**Affiliations:** ^1^Department of Neurology and Psychiatry, Sapienza University of Rome, Rome, Italy; ^2^Neuromed Institute IRCCS, Pozzilli, Isernia, Italy; ^3^St George’s University of London, London, UK

**Keywords:** Parkinson’s disease, bradykinesia, hypomimia, emotion, motor control

## Abstract

**Background:**

Altered emotional processing, including reduced emotion facial expression and defective emotion recognition, has been reported in patients with Parkinson’s disease (PD). However, few studies have objectively investigated facial expression abnormalities in PD using neurophysiological techniques. It is not known whether altered facial expression and recognition in PD are related.

**Objective:**

To investigate possible deficits in facial emotion expression and emotion recognition and their relationship, if any, in patients with PD.

**Methods:**

Eighteen patients with PD and 16 healthy controls were enrolled in this study. Facial expressions of emotion were recorded using a 3D optoelectronic system and analyzed using the facial action coding system. Possible deficits in emotion recognition were assessed using the Ekman test. Participants were assessed in one experimental session. Possible relationship between the kinematic variables of facial emotion expression, the Ekman test scores, and clinical and demographic data in patients were evaluated using the Spearman’s test and multiple regression analysis.

**Results:**

The facial expression of all six basic emotions had slower velocity and lower amplitude in patients in comparison to healthy controls (all *P*s < 0.05). Patients also yielded worse Ekman global score and disgust, sadness, and fear sub-scores than healthy controls (all *P*s < 0.001). Altered facial expression kinematics and emotion recognition deficits were unrelated in patients (all *P*s > 0.05). Finally, no relationship emerged between kinematic variables of facial emotion expression, the Ekman test scores, and clinical and demographic data in patients (all *P*s > 0.05).

**Conclusion:**

The results in this study provide further evidence of altered emotional processing in PD. The lack of any correlation between altered facial emotion expression kinematics and emotion recognition deficits in patients suggests that these abnormalities are mediated by separate pathophysiological mechanisms.

## Introduction

Parkinson’s disease (PD) is a neurodegenerative disease characterized by motor and non-motor symptoms (1, 2). Although the main pathophysiological mechanism of PD is the dopaminergic denervation of the basal ganglia (3, 4), other structures are involved. Indeed, the cingulate cortex and amygdala are also known to be involved in the neurodegenerative process of PD (5–8). Therefore, a proportion of patients suffer from altered emotional processing (9, 10).

Accordingly, spontaneous facial expressivity has consistently been reported to be impaired in PD (11–16). The ability to mimic a facial expression shown in a picture (posed emotions) has been reported to be either normal (16) or impaired (15, 17, 18). However, there is a limited amount of information available regarding the objective assessment of facial expressions of the basic emotions in PD using neurophysiological techniques (16, 19–21). By contrast, facial emotion recognition has been extensively investigated in PD patients (22). Impaired emotion recognition has consistently been reported for disgust (23–26), whereas data on other emotions are less concordant (27–33). One important issue that has yet to be clarified is whether a relationship exists between facial emotion expression abnormalities and facial emotion recognition deficits in PD and whether these two deficits share the same underlying pathophysiological mechanisms (19, 21, 34). This information would provide a deeper insight into the pathophysiology of altered emotional processing in PD patients.

The aim of this study was to investigate whether possible deficits of facial emotion expression in PD patients are related to other aspects of emotional processing, such as emotion recognition. For this purpose, we first kinematically assessed the facial expression of the six basic emotions, i.e., anger, disgust, fear, happiness, sadness, and surprise, in PD patients, and then assessed possible deficits in facial emotion recognition by means of the Ekman test in the same sample of patients. Finally, we investigated the possible relationship between kinematic abnormalities of facial emotion expression, emotion recognition deficits, and clinical or demographic data. Data from PD patients were compared with those obtained from a sample of healthy control subjects.

## Materials and Methods

### Participants

Patients were recruited from the outpatient clinic of the Department of Neurology and Psychiatry, Sapienza University of Rome. Eighteen PD patients were completed the study [seven women, mean age ± 1 SD: 58.8 ± 5.5]. Sixteen healthy subjects (HS) (six women, mean age ± 1SD: 61.2 ± 9.8)—matched for age, gender, and educational level—were included as a control group (Table 1). The diagnosis of idiopathic PD was based on clinical criteria (1, 2, 35). Motor symptoms and disease staging were assessed by means of the movement disorder society-sponsored revision of the Unified Parkinson’s Disease Rating Scale (MDS-UPDRS part III) scale (36–38) and Hoehn & Yahr staging scale (H&Y). Patients were excluded if they had dementia or other neuropsychiatric disturbances (such as depression, anxiety, or psychosis) as defined by means of the DSM-IV criteria; other exclusion criteria were involuntary facial movements and a history of facial trauma or paralysis that might interfere with facial movements. All the participants were right handed, and none of them were taking drugs that act at the central nervous system level other than antiparkinsonian medications for the PD patients. All the patients were stable responders to dopaminergic medication (i.e., levodopa and extended-release dopamine agonists, alone or in combination) and tested at the same time of the day while they were on their usual therapeutic regimen. In PD patients, the total l-DOPA equivalent daily dose (LEDD) was calculated as in Tomlinson et al. (39). We assessed patients in the ON medication state because we did not detect any significant effect of dopaminergic medications on facial movements, including voluntary blinking and posed smiling kinematics, in previous studies (18, 40, 41). All the subjects gave their written informed consent. The study was approved by the local institutional review board, and experiments conformed to the regulations laid down in the Declaration of Helsinki.

**Table 1:** **Demographic and clinical characteristics of participant groups**.

	PD patients	HS	*P*-values
Gender ratio	7F/11M	6F/10M	0.93
Age	58.8 ± 5.5	61.2 ± 9.8	0.54
Education	11.8 ± 4.2	10.8 ± 4.3	0.65
Disease duration	5.9 ± 2.3	–	–
Movement disorder society-sponsored revision of the unified Parkinson’s disease rating scale (part III)	16.9 ± 3.9	–	–
LEDD	394.44 ± 181.31	–	–

*PD, Parkinson’s disease; HS, healthy subjects; F, females; M, males; LEDD, levodopa equivalent daily dose*.

*Age, education, and disease duration are expressed in years. Data given are means ± 1 SD*.

### Kinematic Recordings and Analysis of Facial Expression

Facial movements were recorded using a 3D optoelectronic system (SMART motion system, BTS Engineering, Milan, Italy). This system comprises three infrared cameras (sampling rate, 120 Hz) that follow the 3D displacement of reflective markers taped on the face of the subjects. We used 21 reflective markers of negligible weight. Four markers were placed on the medial and lateral part of the eyebrow (bilaterally). Two markers were placed on the middle of the lower eyelids. Two markers were placed over the nasolabial furrows. Two markers were positioned on the labial corners. Four markers were placed on the medial and lateral part of the superior and inferior lips. Finally, one marker was placed over the chin. Head movement contaminations were avoided by using three additional markers placed over the tip of the nose and over the zygomatic process of the temporal bone, bilaterally. A software (SMART Analyzer, BTS Engineering, Milan, Italy) reconstructed the 3D spatial displacement of the reflective markers off-line and determined the kinematic features of the facial expressions (i.e., the peak velocity and amplitude).

We quantified the facial expressions of emotions using the facial action coding system, which distinguishes 44 different action units (AUs) produced by a single muscle or a combination of muscles (42, 43). For this analysis, we selected and assessed the kinematics of the main and most consistent AUs that reflect each specific emotion (Table 2). In order to generate a single measurement, we also computed composite scores by calculating the average values of the AUs selected for each facial expression of emotion.

**Table 2:** **Kinematic variables of facial emotion expressions in patients with Parkinson’s disease (PD) and in healthy subjects (HS)**.

	Peak velocity			Amplitude		
	PD patients	HS	*P* values	PD patients	HS	*P* values
**Anger**						
Brown lowerer—AU4	23.57 ± 4.51	32.89 ± 4.77	0.097	3.25 ± 0.60	4.69 ± 0.81	0.147
Upper lip raiser—AU10	22.21 ± 3.45	39.74 ± 4.90	0.015*	2.17 ± 0.28	5.76 ± 1.31	0.015*
Jaw drop—AU26	50.07 ± 11.38	131.29 ± 63.41	0.128	7.18 ± 1.41	12.17 ± 2.46	0.078
**Disgust**						
Nose wrinkler—AU9	22.02 ± 4.85	42.50 ± 6.54	0.015*	3.58 ± 0.56	6.68 ± 0.79	0.004*
Lip corner depressor—AU15	23.78 ± 3.83	33.23 ± 9.54	0.427	4.81 ± 0.99	6.87 ± 1.13	0.062
Jaw Drop—AU26	33.83 ± 6.77	52.14 ± 13.46	0.580	7.33 ± 1.51	10.77 ± 2.34	0.254
**Fear**						
Inner brown raiser—AU1	18.15 ± 2.44	35.67 ± 3.57	0.010*	2.30 ± 0.38	3.52 ± 0.35	0.128
Outer brown raiser—AU2	27.58 ± 2.78	47.91 ± 7.00	0.006*	2.80 ± 0.42	4.50 ± 0.45	0.027
Jaw drop—AU26	45.28 ± 9.54	123.42 ± 28.60	0.045	8.75 ± 2.32	14.12 ± 3.68	0.097
**Happiness**						
Cheek raiser—AU6	17.74 ± 4.17	40.44 ± 6.60	0.004*	2.53 ± 0.37	6.03 ± 0.95	<0.001*
Lip corner puller—AU12	30.26 ± 4.76	53.03 ± 7.17	0.005*	5.01 ± 0.57	8.98 ± 1.16	0.005*
**Sadness**						
Brown lowerer—AU4	7.82 ± 1.09	13.91 ± 2.17	0.009*	1.31 ± 0.24	1.97 ± 0.34	0.097
Lip corner depressor—AU15	9.86 ± 1.61	18.57 ± 2.49	0.001*	1.70 ± 0.27	3.36 ± 0.43	0.001*
**Surprise**						
Inner brown raiser—AU1	25.90 ± 3.35	46.14 ± 4.99	0.002*	2.88 ± 0.40	4.04 ± 0.52	0.012
Outer brown raiser—AU2	32.26 ± 3.60	54.26 ± 4.44	0.001*	3.66 ± 0.39	4.86 ± 0.41	0.053
Jaw drop—AU26	60.59 ± 9.07	156.20 ± 41.02	0.008*	10.09 ± 1.95	17.20 ± 4.89	0.090

*Plus and minus values are mean ± 1 SEM. Data of all the action units (AU), except AU26, are pooled from both hemifaces*.

*Statistically significant between group difference.

Facial expressions were studied by asking participants to voluntarily mimic the six basic emotions, i.e., anger, disgust, fear, happiness, sadness, and surprise, so as to achieve the highest expressiveness possible for each emotion. The examiners monitored the correct execution of the task online. Incongruent facial expressions were rejected online.

### Assessment of Emotion Recognition

Emotion recognition deficits were evaluated in all the participants by means of the Ekman 60 Faces test. Pictures of 10 actors’ faces (5 males and 5 females) expressing each of the six basic emotions, i.e., anger, disgust, fear, happiness, sadness, and surprise, were presented on a computer screen one at a time. Participants were asked to identify the emotions represented in the pictures in a six forced-choice response format. Response time was unlimited, but participants were encouraged to respond as quickly as possible. The test yields a score out of a maximum of 60 for the recognition of all six emotions or scores out of 10 for the recognition of each basic emotion.

### Statistical Analysis

Gender differences between PD patients and HS were assessed by means of the Chi-square (χ^2^) test. Possible differences in age and years of education, in the kinematic variables of the main AUs reflecting each specific basic emotion, and in the Ekman test scores (including the global score and the sub-scores of the six basic emotions) between PD patients and HS were evaluated by means of the Mann–Whitney *U*-test. Data from the hemiface corresponding to the more affected side of the body and the hemiface corresponding to the less affected side of the body in patients, and right and left hemiface in HS, were pooled together as no significant difference emerged between the two sides of the faces in a preliminary analysis using the Wilcoxon matched-pairs test (see Results). The peak velocity and amplitude of the AUs reflecting each emotion were assessed in a separate analysis (Table 2).

Possible relationship between the kinematic variables of facial emotion expression and the Ekman test scores were assessed by the Spearman rank order correlation analysis. Subsequently, we also performed a multiple regression analysis using the demographic and clinical data of PD patients, i.e., age, years of education and disease duration, the MDS-UPDRS (part III) global score, hypomimia or bradykinesia items and LEDD, as independent variables and kinematic variables of facial emotion expression, and the Ekman test scores as dependent variables.

Unless otherwise stated, results are indicated as mean values ± 1 SEM. Bonferroni’s correction was applied to multiple comparisons and correlations.

## Results

### Demographic and Clinical Assessments

No significant differences in gender distribution, age, or years of education were observed between PD patients and HS (all *P*s > 0.05, Table 1).

### Facial Emotion Expressions

A preliminary analysis showed no peak velocity and amplitude difference between the hemiface corresponding to the most affected side of the body and the hemiface corresponding to the less affected side of the body in PD patients, and right and left hemiface in HS, for all the AUs examined (all *P*s > 0.05). Data of the two sides of the faces were then pooled together for a subsequent between-group analysis showing no significant difference for the peak velocity and amplitude in various AUs examined between PD patients and HS (Table 2). The analysis revealed that the kinematic variables were overall lower in PD patients than in healthy controls for all the six basic emotions analyzed (Table 2). To sum up, when compared with HS, PD patients displayed an overall and aspecific reduction in facial motor activation during facial emotion expression (Table 2).

### Facial Emotion Recognition

**Figure 1:**
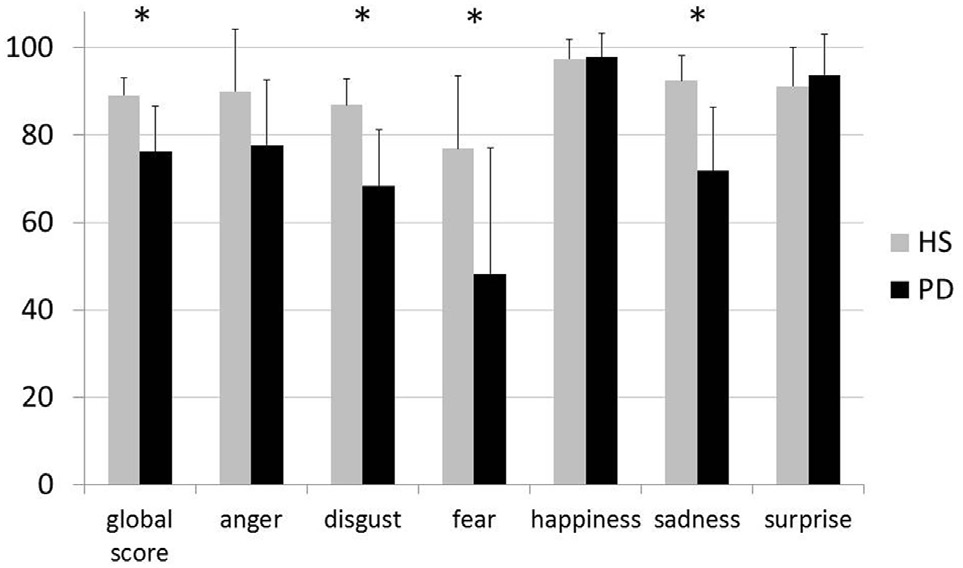
**Results of the Ekman 60 Faces test in healthy subjects—gray histograms and in patients with Parkinson’s disease—black histograms**. The *X* axis indicates the global score and sub-scores for the six basic emotions during the Ekman test; the *Y* axis indicates the percentage of correct responses in each group. Vertical bars indicate 1 SD. Asterisks indicate statistically significant between group difference.

The overall Ekman score of the PD group was significantly lower than that of the HS (*P* < 0.001). The analysis yielded significantly lower scores for the sub-scores of disgust, fear, and sadness in PD patients than in HS (all *P* < 0.001) and a difference approaching significance between the two groups for the sub-score of anger (*P* = 0.01, corrected alpha level 0.008). No differences were detected between the two groups in the recognition of happiness and surprise (both *P* ≥ 0.05). To sum up, when compared with HS, PD patients displayed an overall reduction in emotion recognition, due to a specific deficit in the recognition of disgust, sadness, and fear (Figure 1).

### Relationship between Kinematic Data, Ekman Test Scores, and Demographic and Clinical Data

The Spearman rank order correlation analysis showed no relationship between the composite scores of both peak velocity and amplitude for the six basic emotions, i.e., anger, disgust, fear, happiness, sadness, and surprise, and the emotion recognition deficits in PD patients (all *P*s > 0.05). Multiple regression analysis detected no relationship between demographic and clinical data, i.e., age, years of education and disease duration (in years), as well as motor symptom severity, i.e., the MDS-UPDRS (part III) global score, hypomimia or bradykinesia items, and LEDD, and kinematic data or Ekman test scores (all *P*s > 0.05).

## Discussion

The results of this study indicate that facial expression of anger, disgust, fear, happiness, sadness, and surprise and recognition of disgust, sadness, and fear are impaired in PD patients. We did not detect any correlation between facial expression kinematic abnormalities and emotion recognition deficits in patients, which suggests that different pathophysiological mechanisms underlie these two abnormalities.

There may be several reasons for deficits in facial emotion expression and recognition in PD patients, such as dementia, neuropsychiatric disorders, and other diseases of the facial region, including dyskinesia and facial palsy. However, we ruled out the possibility that they might have affected the results in our patients by including these conditions among the exclusion criteria. Since we also excluded patients treated with psychotropic medications (other than dopaminergic medications), we also rule out the possibility that impaired emotion expression and recognition are due to aspecific numbing or blunting, which are common side effects of several drugs that act at the level of the central nervous system. Finally, since the demographic and clinical data of the PD patients and HS were similar, we also rule out any confounding effect of these features.

The results of the kinematic evaluation of the facial expressions of anger, disgust, fear, happiness, sadness, and surprise in PD patients compared with HS point to an overall, aspecific slowness, i.e., bradykinesia, of facial emotion expression as assessed by the kinematic analysis of specific AUs of each basic emotion. The results also extend the observations reported in previous studies on PD patients by revealing kinematic abnormalities in spontaneous and posed smiling (13, 14, 18), as well as those of more recent studies on facial mimicry deficits, as assessed by EMG (19, 21). Voluntary facial movements, including expressions of emotions, are primarily mediated by primary and non-primary motor cortical areas in the frontal lobe through direct corticobulbar projections to the facial motor nucleus (11, 44). In particular, the motor areas in the medial wall of the frontal lobe, including the supplementary motor area and cingulate cortex, are involved in generating voluntary movements associated with emotional expression (45). Our findings support the hypothesis that in PD a basal ganglia dysfunction might interfere with both primary and non-primary motor area activation in the frontal lobe, thereby resulting in an aspecific slowness of the facial expression of emotion.

In this study, we found that movement slowness during all the facial expressions of emotions affected both the upper and lower hemifaces. These results, which are in keeping with previous findings, indicate that muscle hypoactivation and slower movements of the lower face in patients with PD than in HS during the expression of facial emotions (18, 19, 21). By contrast, the results of previous studies have shown that the ability of PD patients to voluntarily perform stereotyped movements, such as blinking, is relatively well preserved, there being no evidence of altered velocities or amplitudes (40, 41, 46). Since the muscles of the peri-orbital region (upper face), particularly those of the eyelid, are characterized by low inertia and are innervated bilaterally, they are less likely to be impaired than the muscles in the mouth area (lower face), which are cross-innervated prevalently from the contralateral side (11, 44, 45). Another explanation is that complex facial movements that require the integration of afferent influences from limbic structures (9, 11), such as emotion expressions, are more susceptible to impairment than more stereotyped movements, such as blinking.

Clinical observations indicate that more severe facial expression deficits, i.e., hemihypomimia, may be observed to the most affected side of the body and to the side of the body on which symptoms start in a low proportion of patients with PD (47, 48). We did not observe any differences in the kinematic features of the six basic emotions between the left and right hemifaces in healthy controls, or between the hemiface ipsilateral or contralateral to the most severely affected side of the body in PD patients. The present findings extend previous clinical observations and kinematic findings based on the evaluation of posed smiling (18) and suggest that there is no significant hemiface difference in facial motor activation in the majority of PD patients.

An innovative feature of this study is the evaluation we performed to investigate whether any relationship exists between deficits in facial emotion expression (by means of the kinematic analysis) and emotion recognition to determine whether these abnormalities are related to altered connectivity between the emotion-related activation of limbic structures and downstream motor effectors. In our PD sample, we detected kinematic abnormalities in all six basic emotions, whereas emotion recognition deficits were observed specifically for disgust, fear, and sadness. Moreover, we did not detect any correlation between individual deficits in emotion expression and recognition. Our results thus indicate that these two abnormalities that reflect altered emotional processing in PD, i.e., facial emotion expression and emotion recognition, are probably mediated by different pathophysiological mechanisms. Indeed, the impaired expression of emotions in PD is likely to reflect impaired basal ganglia loops and abnormal primary motor and pre-motor area activation in the frontal lobe. By contrast, the specific impairment of emotion recognition in PD is likely to reflect a predominant involvement of limbic system components, including the insula and amygdala (5–9). As an alternative explanation, components that regulate facial emotion expression other than pure neuromuscular activation, as measured by the kinematic analysis, may be related to the recognition of facial emotions expressed by others (49).

We acknowledge that our study has certain limitations. First, PD is a clinically heterogeneous disorder with different subtypes that may be associated with different cognitive and emotional correlates; it is thus possible that the different disorder subtypes have different impacts on specific deficits of emotional processing. Second, since we only enrolled PD patients whose symptom severity was moderate, we do not know whether our results apply to other stages of the disease; it is possible that facial emotion expression and recognition in the earlier or more advanced disease stages differ from those that we observed. Future studies should thoroughly assess patients in the various stages of the disease to clarify this issue. Owing to the relatively small sample size, we did not specifically investigate the influence of other factors such as gender or age nor did we investigate the possible effect of dopaminergic medication because we previously observed that dopaminergic medication does not significantly affect voluntary facial movements. Finally, it should be borne in mind that we decided to examine only mimicked facial emotion expressions and not posed emotions using the same pictures of the emotion recognition task, and moreover, we assessed facial emotions in an experimental setting, without considering spontaneous facial emotion expressions, whose pathophysiology may differ from that of the mimicked expressions.

## Conclusion

This study provides novel information on altered emotional processing in PD. To the best of our knowledge, this is the first study that has examined facial expression kinematic abnormalities of all the primary emotions and facial recognition abnormalities in the same sample of patients with PD. The different patterns of impairment observed between facial emotion expression and emotion recognition in PD, as well as the lack of any correlation between the two abnormalities, point to different underlying pathophysiological mechanisms. Both emotion expression and recognition abnormalities should thoroughly be assessed in PD because they may worsen social interaction and the quality of life in PD patients.

## Ethics Statement

Institutional Review Board, Sapienza University of Rome, Rome, Italy.

## Author Contributions

MB, IB, GP, and LM: conception of the work; acquisition, analysis, and interpretation of data; and drafting the work. LR, GF, and AB: conception of the work; analysis and interpretation of data; and revising the work critically for important intellectual content.

## Conflict of Interest Statement

The research was conducted in the absence of any commercial or financial relationships that could be construed as a potential conflict of interest. The reviewers GP and FN and handling Editor declared their shared affiliation, and the handling Editor states that the process nevertheless met the standards of a fair and objective review.

## Abbreviations

AUs, action units; FACS, facial action coding system; H&Y, Hoehn & Yahr scale; MDS-UPDRS, movement disorder society-sponsored revision of the unified Parkinson’s disease rating scale; PD, Parkinson’s disease.
